# Large-Scale Comparative Genomic Ranking of Taxonomically Restricted Genes (TRGs) in Bacterial and Archaeal Genomes

**DOI:** 10.1371/journal.pone.0000324

**Published:** 2007-03-28

**Authors:** Gareth A. Wilson, Edward J. Feil, Andrew K. Lilley, Dawn Field

**Affiliations:** 1 Centre for Ecology and Hydrology (CEH) Oxford, Oxford, United Kindgom; 2 Department of Biology and Biochemistry, University of Bath, Bath, United Kingdom; Pasteur Institute, France

## Abstract

**Background:**

Lineage-specific, or taxonomically restricted genes (TRGs), especially those that are species and strain-specific, are of special interest because they are expected to play a role in defining exclusive ecological adaptations to particular niches. Despite this, they are relatively poorly studied and little understood, in large part because many are still orphans or only have homologues in very closely related isolates. This lack of homology confounds attempts to establish the likelihood that a hypothetical gene is expressed and, if so, to determine the putative function of the protein.

**Methodology/Principal Findings:**

We have developed “QIPP” (“Quality Index for Predicted Proteins”), an index that scores the “quality” of a protein based on non-homology-based criteria. QIPP can be used to assign a value between zero and one to any protein based on comparing its features to other proteins in a given genome. We have used QIPP to rank the predicted proteins in the proteomes of Bacteria and Archaea. This ranking reveals that there is a large amount of variation in QIPP scores, and identifies many high-scoring orphans as potentially “authentic” (expressed) orphans. There are significant differences in the distributions of QIPP scores between orphan and non-orphan genes for many genomes and a trend for less well-conserved genes to have lower QIPP scores.

**Conclusions:**

The implication of this work is that QIPP scores can be used to further annotate predicted proteins with information that is independent of homology. Such information can be used to prioritize candidates for further analysis. Data generated for this study can be found in the *OrphanMine* at http://www.genomics.ceh.ac.uk/orphan_mine.

## Introduction

The availability of hundreds of complete bacterial genome sequences has made it possible to explore how the evolutionary diversification of gene content reflects the ecological needs and opportunities of different taxa. It is well known that the gene content of bacterial and archaeal genomes can vary widely and that only a very few genes are truly universal [1]–[3]. As a consequence, genes can differ significantly in their taxonomic distributions, with more broadly conserved genes having ‘housekeeping’ functions and less conserved genes being responsible for the phenotypic differences observed between organisms. Lineage-specific, or “taxonomically restricted” genes (TRGs), are defined as being exclusively restricted to a particular taxonomic group [4]. In such a framework, genes may be TRGs at any taxonomic level (*i.e.* domain-, family, genus-, species- or strain-specific). TRGs at the species and strain-levels are of most interest in the search for genotypes which help define exclusive ecological adaptations to particular niches.

The study of narrowly distributed TRG's is confounded by the fact that many are short, repetitive or have unusual A+T contents [5], and the assumption that many such short coding sequences (CDS) represent annotation errors [6]. Over-annotation of genomes, resulting in an excess of small predicted proteins is clearly evident in certain genomes (*e.g* the initial annotation of *Aeropyrum pernix* [7]) and is proposed to be an unfortunate feature of many genomic annotations [6], [8], [9]. This overannotation could mask intergenic regions containing small non-coding RNAs. It is also possible that many TRGs remain ‘orphaned’ for no other reason than the sampling bias in public genome databases [10]. It is well-known that the current collection is highly biased towards certain organisms (most notably pathogens, γ-Proteobacteria, and Firmicutes) [11]. This results in the trend that taxonomic isolation is correlated with an increased percentage of orphans [8]. It is therefore expected that homologues for many orphan predicted proteins in taxonomically isolated lineages that lack close relatives in genomic databases will be found once the taxonomic gaps in the genomic database begin to be filled [10].

Despite potential errors in our current estimation of the numbers and identities of narrowly distributed TRGs, there is growing evidence that many, including those that are currently orphaned, are of biological significance. Hence there is a growing need to untangle erroneous CDS from authentic species- and strain-level TRGs [12]–[14]. Dispersed examples of the latter are most frequently found as the result of in depth *in silico* [5] or empirical studies [12] of a particular organism or small group of organisms. Increasingly, examples are being identified as the result of whole genome sequencing [14]. One example to come from complete genome sequencing is the TCP virulence locus of *Vibrio cholerae* Tor N16961. Once a cluster of largely orphaned CDS, a homologous region has recently been found in the squid symbiont *Vibrio fischeri* [15]. The TCP genes code for the toxin co-regulated pili in *V. cholerae* and serve as its critical intestinal colonisation factor, providing the receptor for entry of the temperate filamentous phage CTX^Φ^, which contains the cholera toxin genes, *ctxAB* [16] into the cell [17]. Likewise, the sequencing of many genomes is confirming the presence of many strain-specific genes which form the “pan-genome” of many species [18], [19].

Given the potential significance of orphaned and narrow-range TRGs and the confounding sources of error associated with currently annotated genomes, it is clear that a reliable objective measure of the potential ‘quality’ of a given CDS would be useful [See 20 for the scoring of CDS with homology]. This could be used to prioritize it either as a candidate for further characterization or as an error. Motivated by this requirement, and with a specific focus on orphans and narrow-range TRGs, we have devised a scoring system that allows the ‘ranking’ of predicted proteins based on a variety of features, reflecting the likelihood that a given CDS encodes a protein.

We previously reported that the absolute number of single-copy TRGs from the complete and published genomes of Bacteria and Archaea is increasing [4]. The most phylogenetically and ecologically unique species contribute the most unique genes, in part due to undersampling of these genetic lineages [4]. For that study we generated two datasets. The first contained all orphans as defined by BLAST (using a threshold of 10^−3^), the second applied an arbitrary length cutoff of ≥150 amino acids and excluded all CDS with low complexity (highly repetitive) regions to remove likely CDS enriched in artefacts. The method of scoring CDS described here extends this ‘selective filtering’ approach and is called the ‘Quality Index for Predicted Proteins’ (QIPP). We describe the use of QIPP as it is applied to the reanalysis of this data set based on the inclusion of five criteria selected for their presumed ability to detect purifying selection and CDS which are unlikely to occur by chance alone. These are length [6], percentage low complexity (a measure of the degree of repetition) [21], difference in G+C composition of sequence and genome [22], average amino acid cost [23], [24] and neighbourhood distribution (ND) [25].

## Results

### The orphan and non-orphan components of many proteomes have different overall characteristics

**Figure 1:**
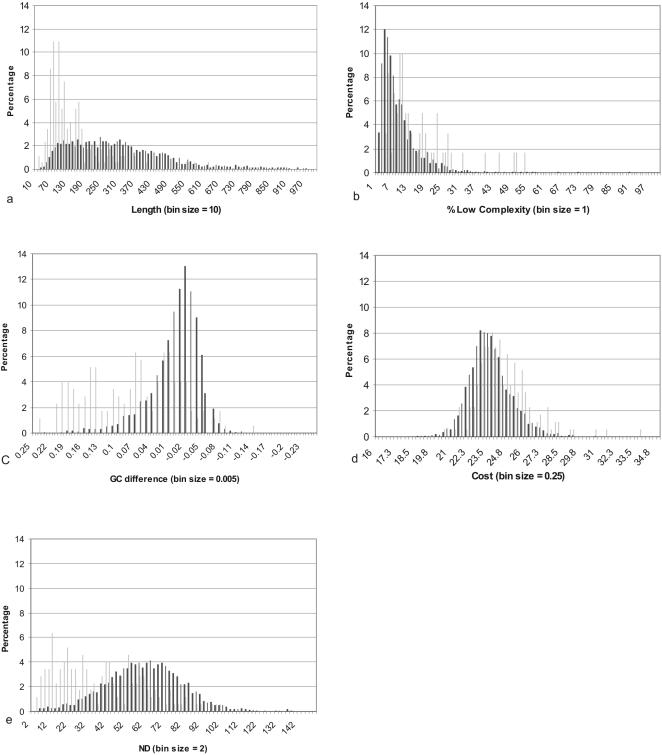
Distributions of orphans and non-orphans in E.coli K12. The predicted proteins in E.coli K12 that were found to be unique (light gray) when compared to 122 bacterial proteomes (shown in Table S1) were designated as orphans (n = 174). All remaining proteins (dark gray) were non-orphans (n = 4137). Distributions of values for both groups were calculated as a percentage for (a) length, (b) percent low complexity, (c) G+C difference from the mean, (d) Cost and (e) Neighbourhood Distribution.

To examine whether orphaned CDS, which are expected to be on average smaller [6] and more A+T rich [5], [26] have significantly different QIPP scores than non-orphans, we re-examined our original data set [4]. QIPP scores were calculated for each protein in this data set of 122 proteomes [4] as described in the Materials & Methods. In total, the distributions of all five criteria (length, low complexity, G+C content, amino acid cost and neighbourhood distribution (Table 1)) differ significantly between orphans and non-orphans in 61 of the 122 species examined (p<0.05, Mann-Whitney). 3 or more criteria are significant in 117/122 species. Four of the remaining five species contained fewer than 10 orphans, and when all such genomes (n = 6) were excluded 115 of the remaining 116 species had orphans that differed significantly from the non-orphans for three or more criteria. The strikingly different values for *Escherichia coli* K12 can be seen in Figure 1 as an example of these trends. The distribution of the QIPP scores for orphan and non-orphan TRG's were found to be significantly different for 119 of the 122 genomes (p<0.05, Mann-Whitney). The remaining three genomes contained 2 or less orphans and thus could not provide significant discriminatory power. Overall, the QIPP scores for all orphan (mean = 0.38, +0.14) and non-orphan (mean = 0.54, +0.14) TRG's were significantly different (p = 0.000, Mann-Whitney). These results confirm that the criteria used for the QIPP scores can reliably distinguish between “orphan-like” (less well conserved) and “non-orphan-like” (more widely conserved) genes.

**Table 1:** Criteria used for the calculation of QIPP

Optimality Criteria	Desirable Values	Ranked by
Length	Long	Distribution of absolute lengths of non-orphans
Complexity	Complex	Distribution of percent low complexity in non-orphans
Cost	Low	Distribution of the average cost per amino acid of non-orphans
G+C Composition	Average composition	Distribution of the difference in G+C content of non-orphans and the genome G+C compostion
Neighbourhood Distribution	Location among genes with a broad distribution	Average of the number of genomes with homologues to the 5 genes flanking either side of a gene.

### Ranking orphan CDS using QIPP scores

**Figure 2:**
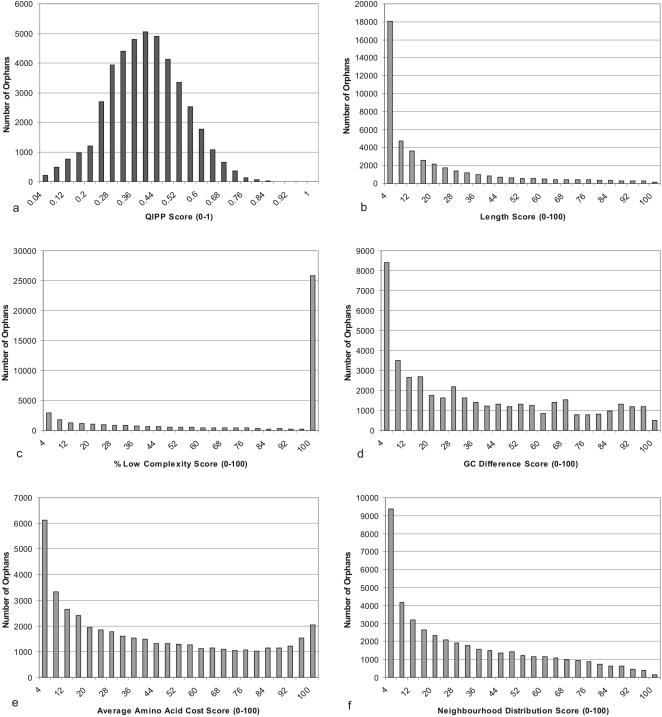
QIPP and Criterion Distributions of orphans in 122 bacterial genomes. The orphans (n = 43513) obtained from 122 bacterial genomes were scored and the distribution plotted according to (a) QIPP and the individual criteria that constitute QIPP: (b) length, (c) percent low complexity, (d) G+C difference from the mean, (e) cost and (f) Neighbourhood Distribution.

The distribution of QIPP scores across the orphans in this data set was examined to determine if there was sufficient variation to rank them. Figure 2a shows that QIPP scores range from 0.0 to 0.9 (out of a possible range from zero to one) and so the index does have discriminatory power. The overall QIPP scores for each proteome deviate from the normal distribution for all five reference genomes, with too few high-scoring CDS and a longer than expected left-hand tail of low-scoring proteins (Darling-Anderson p<.005). This is due to the fact that for each criterion (with the exception of low complexity) there are few proteins with very high ranks (Figure 2b–f).

We then examined the quality of the highest-scoring orphans to see if our list contained a significant number of potentially ‘authentic’ orphans–i.e. those unlikely to occur by chance. The extreme right hand distribution of these QIPP scores contains a total of 2,010 single-copy TRGs (≥95^th^ percentile with a minimum score of 0.62), 1,260 are longer than 200 amino acids, a criterion that when used in isolation, is generally accepted to signify ‘authentic’ CDS [6]. Relaxing the QIPP score threshold, and using only length as a criterion, a total of 9858 (22.66%) single-copy TRGs are found in this data set which are ≥200 amino acids. A subset of these, 2,445 (5.62%), are ≥400 amino acids.

When interpreting the origins of such high-quality single-copy TRGs, the taxonomic uniqueness of each parent genome must be considered. Of those with QIPP scores above the 95th percentile (> = 0.62), only 467 (23%) are from 62 species (8 per genome) sampled down to the species level (*i.e.* another species from the same genus is available in the data set) (average QIPP score = 0.66). In contrast, 1,543 (77%) originate from 60 species which only have more distant relatives in this data set. It is presumed that these genomes include many TRGs exclusive to higher taxonomic levels; 24 genomes are unique at the genus level (259 orphans, 11 per genome, average QIPP score = 0.66), 30 at the family level (931 orphans, 31 per genome, average QIPP score = 0.67) and 6 at the division level (353 orphans, 59 per genome, average QIPP score = 0.67). Of those larger than 200 amino acids, 2,878 (29%) are from 62 species (46 per genome) sampled down to the species with an average QIPP score of 0.43. The remaining 6,980 (71%), originate from 60 species unique at the genus level (1,439 total, 60 orphans per genome, average QIPP score = 0.44), the family level (4,263 total, 142 orphans per genome, average QIPP score = 0.48) and the division level (1,278 total, 213 orphans per genome, average QIPP score = 0.50).

**Figure 3:**
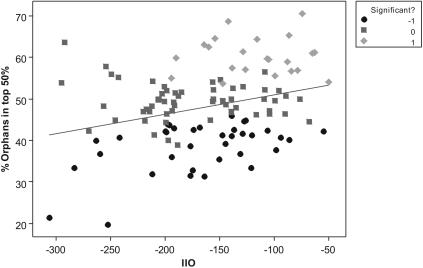
Genomes which are more taxonomically isolated have larger numbers of high-scoring orphan predicted proteins. Chi-squared tests were used to determine which genomes had significantly more predicted proteins in the top 50% of the list of ranked orphan predicted proteins than would be expected by chance (−1 = significantly less orphans than expected in top 50% rank, 0 = no significant difference and 1 = significantly more orphans than expected in top 50% rank).

When plotted against genetic similarity, more distantly related genomes contribute on average more high-quality, single-copy TRGs (Table S1 and Figure S1). Chi-squared tests were used to identify genomes that made a greater contribution than expected to the top 50% of the ranked list (Figure 3). Genomes that did not contain enough orphans (>5) to perform a chi-squared test were removed from the analysis (n = 6). Genomes that contribute more high ranking QIPP scores are more distantly related (Figure 3, Anova p = 0.000) but only a low proportion of variability in top-ranking scores is explained by a regression analysis (p = 0.000, R-squared = 10.63%).

### Less conserved genes have lower QIPP scores

**Figure 4:**
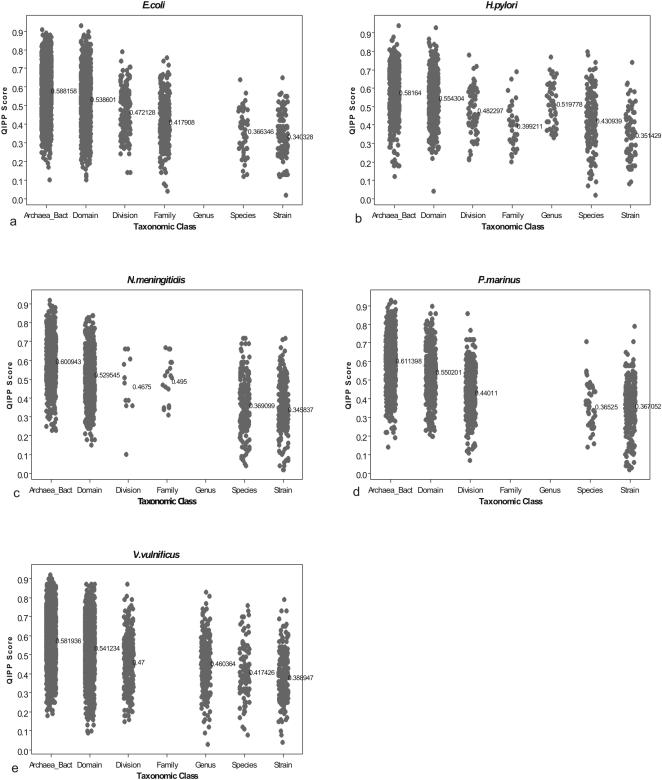
Calculated QIPP scores for 5 bacterial genomes split into taxonomic classes. Every predicted protein in (a) *E.coli* K12, (b) *H.pylori* 26695, (c) *N.meningitides* MC58, (d) *P.marinus* CCMP1375 and (e) *V.vulnificus* CMCP6 was put into the taxonomic level at which it was restricted and scored according to QIPP.

The difference between orphan and non-orphan QIPP scores suggests that it might be possible to predict *a priori* how conserved a particular CDS might be using QIPP scores in the absence of homology. To explore this further, we selected a subset of five reference genomes from the best-sampled taxa in our original dataset for which intra-specific comparisons yielding high numbers of strain-specific orphans were also available (Table 2). For each reference genome the taxonomic distribution of all predicted proteins at the Archaea/Bacteria level, domain, division, family, genus, species and strain level (Figure 4) was determined.

**Table 2:** Numbers and percentages of species-specific and strain-specific genes after the addition of a second strain in five bacterial species.

Reference Genome	Second Genome	Orphan genes (N = 122)	Species-specific (N = 122+1)	Strain-specific (orphan genes) (N = 122+1)
*Escherichia coli K12* (NC_000913)	*Escherichia coli UPEC-CFT073* (NC_004431)	174	52 (29.89%)	122 (70.11%)
*Helicobacter pylori 26695* (NC_000915)	*Helicobacter pylori J99* (NC_000921)	258	181 (70.16%)	77 (29.84%)
*Neisseria meningitides MC58* (NC_003112)	*Neisseria meningitides Z2491* (NC_003116)	431	222 (51.51%)	209 (48.49%)
*Prochlorococcus marinus CCMP1375* (NC_005042)	*Prochlorococcus marinus MIT9313* (NC_005071)	291	40 (13.75%)	251 (86.25%)
*Vibrio vulnificus CMCP6* (NC_004459,NC_004560)	*Vibrio vulnificus YJ016* (NC_005139,NC_005140)	348	101 (29.02%)	247 (70.98%)

The average QIPP scores and percentages of predicted proteins exclusive to each of these taxonomic levels are given in Table 3. Overall, average scores are relatively uniform across the five genomes at each of the 7 taxonomic levels examined. Scores range from an average of 0.60 for proteins conserved across bacteria and archaea down to 0.35 for proteins conserved at the strain-level. These average scores are significantly different across TRG's exclusive to different taxonomic levels (Anova, p = 0.000 for every genome). The data show an overall decrease in QIPP score as the degree of conservation narrows (Figure 4). For the five genomes, when all CDS are taken into account, a regression analysis provides a p-value of 0.000 with R-squared values ranging from 20.3% to 36.3%.

**Table 3:** Table showing the average QIPP score for predicted proteins at each taxonomic level for five selected bacterial genomes.

	Bacteria/Archaea	Bacterial Domain	Division	Family	Genus	Species	Strain	1^st^–3^rd^ Quartile Range
*E.coli* K12	0.59 (47.75)	0.54 (36.31)	0.47 (4.36)	0.42 (7.54)	N/A	0.37 (1.21)	0.34 (2.83)	0.45–0.65
*H.pylori* 26695	0.58 (43.04)	0.55 (30.58)	0.48 (4.70)	0.40 (2.42)	0.52 (2.86)	0.43 (11.51)	0.35 (4.90)	0.44–0.64
*N.meningitidis* MC58	0.60 (41.85)	0.53 (35.98)	0.47 (0.58)	0.5 (0.87)	N/A	0.37 (10.68)	0.35 (10.05)	0.42–0.64
*P.marinus CCMP1375*	0.61 (44.10)	0.55 (21.15)	0.44 (19.29)	N/A	N/A	0.37 (2.13)	0.37 (13.34)	0.42–0.65
*V.vulnificus CMCP6*	0.58 (44.06)	0.54 (36.96)	0.47 (6.46)	N/A	0.46 (4.85)	0.42 (2.23)	0.39 (5.44)	0.44–0.64

The numbers in brackets show the percentage of proteins in that genome at that taxonomic level. Scores are highest for proteins which are most highly conserved and decrease across taxonomic categories. N/A = genome not available for comparison. The final column shows the values for the CDS of the 2 quartiles around the median QIPP score in each of the five genomes.

The differences in mean QIPP scores between different groups of TRG's are largest for comparisons between groups of CDS conserved above the level of division and those conserved at the species- and strain-level (Table 3). Still, average QIPP scores, are significantly different between all higher TRG groups when compared to the average for species-level TRGs, while groups of species- and strain-level TRGs cannot be distinguished (Table 4). Interestingly, scores from the gene prediction software Glimmer could be used to separate only 7 of the 15 comparisons presented in Table 4. Hence QIPP provides additional information which is useful for post-processing gene predictions such as those made by Glimmer, in the absence of homology.

**Table 4:** Statistical significance of QIPP (Q) and Glimmer (G) scores when differentiating between species-specific genes and a respective taxonomic rank.

	Bacteria/Archaea		Bacterial Domain		Division		Family		Genus		Species	
	Q	G	Q	G	Q	G	Q	G	Q	G	Q	G
*E. coli*	***	***	***	***	***		**		N/A	N/A		
*H. pylori*	***	***	***	***	***		*	**	***	***	*	
*N. meningitidis*	***	***	***	***	**		**		N/A	N/A		
*P. marinus*	***	***	***	***	***	***	N/A	N/A	N/A	N/A		
*V. vulnificus*	***	***	***	***	***		N/A	N/A	***			

*** = p< = 0.001, ** = p< = 0.01, * = p< = 0.05 , N/A = No representative genomes at that taxonomic level.

In addition to using QIPP to rank individual CDS, we also investigated whether the data had biological meaning. Using quartile analysis, 50% of the CDS in each of these genomes fall uniformly between the absolute values of 0.43 and 0.64 (Table 3), suggesting rule of thumb cut-offs for QIPP scores associated with the least (below 0.43) and most (above 0.64) highly conserved CDS in a genome. The data further suggest that the most extreme values of QIPP have the highest degree of predictive power for level of conservation (Figure 4). For example, using a minimum threshold score of ≥0.8, 98% of all CDS are members of the most conserved gene families (above the division-level). A total of 58% of CDS with scores less than 0.2 are species- and strain-specific TRGs.

To observe the range of QIPP scores that might be expected from the most highly conserved CDS we examined a subset of universally conserved genes [3]. We found the homologues of these 31 previously defined protein families [3] in the *E. coli* K12 genome and examined their QIPP scores. These QIPP scores range from 0.5 to 0.87 with a mean of 0.69 (±0.099). A large number of these proteins are ribosomal proteins, which are all of shorter than average size for *E. coli*. QIPP score is very poorly correlated with the overall length of these proteins (R-squared = 0.012) suggesting that QIPP is not overly sensitive to any one component criterion. The two highest-scoring proteins, both with a QIPP score of 0.89, are extremely different in length (1,138 for the DNA-directed RNA polymerase, beta subunit versus 323 for the DNA-directed RNA polymerase, alpha subunit). When length is removed as a component criterion of QIPP, the scores of the shortest proteins increase by up to 0.16, while those of the very longest proteins decrease by a maximum of 0.09 giving a new mean value of 0.75 (±0.141).

### Validation of orphans with low QIPP scores using results from transcriptomic and proteomic studies

To test whether we could validate the expression of orphans with low QIPP scores in a well-studied model organism, we searched the MicrobesOnline database [27] for *E.coli* K12 orphans identified in this study. This database provides experimental microarray results for this organism for four stress conditions: heat shock [28], pH [29], UV exposure [30] and tryptophan metabolism [31]. We examined the fifty highest and lowest ranked species-level TRGs (N = 100). The scores of the top ranking CDS ranged from 0.41–0.64 and the bottom from 0.02–0.28. To illustrate the range of CDS involved, the top scoring CDS was 547 amino acids in length, zero percent low complexity, average G+C content, but was more costly than average and came from a poorly characterized region of the genome. By contrast, the CDS with the lowest score of 0.02 was only 60 amino acids in length, 35% low complexity, had a highly deviant base composition, it was also more costly than average and was found in a poorly characterized region of the genome. Of these 100 orphans, 17 had identifiers not found in the MicrobesOnline database and were excluded. Of the remaining 83, only one failed to show any change in expression levels in any of these experiments. In total there were 46 occasions (involving 35 of these 100 orphans) when one of these orphans was included in the list of the 200 proteins reported in Microbes Online showing the largest (up or down) fold change in expression in one of these experiments. Of particular interest was the pH stress experiment where 12 (three in the top and nine from the bottom 50) of the top 100 up-regulated genes were orphans (p = 0.00, chi-square).

*E.coli* K12 proteomic data sets [32]–[34] were also searched. When combined these investigations identified approximately 1,800 expressed proteins. While mRNA was found for 64 of the 174 CDS in *E. coli*, only 4 proteins could be identified for all 174 single-copy TRGs in this data set. These four CDS had an average QIPP score of 0.32 compared to mean score of 0.35 for all *E.coli* orphans.

## Discussion

We have developed an index called “QIPP” (“Quality Index for Predicted Proteins”) which can be used to assign a value between zero and one for a CDS compared to the rest of the genome on the basis of a set of selective criteria. This provides an objective measure of the probability that a given CDS either encodes a protein or is an annotation artefact (incorrect). Very long CDS, with typical nucleotide and amino-acid compositions, no low complexity regions, and which are found in well conserved regions have the highest QIPP scores and are considered most likely to encode proteins.

The distributions of QIPP scores, and trends in the component variables, confirm that orphans show consistent differences when compared with well characterised protein-coding genes *i.e.* they are short, repetitive, possess atypical G+C content, have high average cost for amino acids and are located in poorly characterised regions of the genome. The significant differences in the distributions of QIPP scores between orphan genes and non-orphan genes confirms that QIPP scores represent a valid means to rationalise and automate the identification of those CDS most likely to encode proteins (and find homologues among other available sequences). Because orphans generally have low QIPP scores it is also possible to meaningfully rank them as a subset of all CDS, selectively filter for high-scoring ‘authentic’ orphans, and begin to address the issue of correcting for the high percentage of orphans in current databases that are simply an artefact of sampling bias.

Our data show that the lowest-scoring CDS encode the least evolutionary conserved proteins, *i.e*. those orphans restricted to single strains or species. As such, this approach can also provide evidence on the likely taxonomic range of a CDS in the absence of any useful homology. This is particularly significant given the unrepresentative sampling of the current genomic databases. Low-scoring, taxonomically restricted orphans are most likely to be annotation artefacts: we tested this in the case of *E. coli* K12 by reference to online transcriptomic and proteomic expression data. Surprisingly, these data revealed that even these low-scoring CDS are potentially expressed (given the caveats associated with using microarray data to validate orphans [6] and the fact that *E.* coli is one of the most thoroughly studied organisms) and therefore suggest that annotation artefacts may not be as common as previously suspected. It is clear that empirical validation of genomic annotations is necessary and should be of the highest priority [35]–[37]. At a minimum it would appear premature to dismiss all very low-scoring orphans as having little biological relevance without further evidence.

In effect, the criteria used in the QIPP score reflect the extent of purifying selection acting upon a sequence, which, in the absence of homology, preclude the use of more widely-used methods such as examination of dN/dS ratios [38]. Purifying selection should over time preferentially purge substitutions leading to the use of more metabolically costly amino-acids [39]. Similarly, mutation pressure tends to move in the direction GC->AT rather than *vice versa* [40], [41] and AT enrichment has commonly been cited as a footprint for relaxed or inefficient purifying selection (but see [42]). This can explain the high AT content of obligate endosymbionts or intracellular parasites which are adapted to a restricted niche, undergo restricted gene exchange, and possibly mutate at a high rate due to the loss of DNA repair genes [43]. It is also well documented that phage and other mobile elements tend to show a higher AT content than the host bacterial genome [5], [39]. As highly conserved proteins are likely to encode essential housekeeping functions, and therefore be subject to high levels of purifying selection, the noted correlation between the taxonomic range and QIPP score can be explained within this selective framework. This phenomenon also provides further validation for the use of the QIPP score in identifying “real” genes, as it is expected that CDS which are simply annotation artefacts should be evolving neutrally and hence have very low QIPP scores.

This analysis provides proof of principal that the combined use of different criteria can be a powerful approach to determining the biological relevance of putative CDS. The power of the QIPP score could be improved by the use of additional criteria which are likely to reflect purifying selection, such as codon bias, for example. It is acknowledged that the criteria presently used are unlikely to be independent, and multivariate analysis is required to determine the interactions between the variables and to put corrections in place to improve the predictive power of the index. Preliminary analysis on five reference genomes has revealed a significant correlation (p< = 0.05) between sequence length and complexity, with longer proteins showing more low complexity regions. Further, a significant correlation between G+C content and amino-acid cost was noted in four out of five genomes (the exception being *V. vulnificus*; data not shown).

There is a growing need for metrics that offer a deeper understanding of the detailed content of genomes, especially now that we have such large numbers [44]. QIPP provides such a metric and can be used in combination with other *in silico* methods that can now be used to sift out potentially authentic orphans and improve genomic annotation. Such complementary methods include the analysis and removal of short CDS [6], gene fragments [45], and pseudogenes [8] and the ranking of CDS based on the availability of homology-based information [20]. Integration of the information from such studies would provide the foundation for a single, global list of uncharacterized predicted proteins that could be used to systematically subject them to further *in silico* examination [20], [35], [37]. This data set could further be integrated with empirical evidence from a range of experimental studies, especially high throughput ‘omic studies, as is the case for databases like STRING [46]. *In silico* studies of predicted proteins can help identify candidates for further examination, but any validation of the biological relevance of a particular protein must be based on empirical evidence [13], [35], [47], [48]. In order to comply with the principle of the transparent access to data for the sake of integration [49], all of the data generated in this study is available online in a searchable database, the *OrphanMine*, a database that supports wide-scale downloads of data, including lists of CDS with rich annotations in GFF3 (Generic Feature Format Version 3) (http://song.sourceforge.net/gff3.shtml) format.

In conclusion, the QIPP index supports an objective rationale for prioritising predicted genes for further study, including ‘authentic’ single-copy TRGs. Although further work is required to refine the approach, this represents an important step in the standardisation and automation of identifying biologically important genes in the absence of homology.

## Material and Methods

### Processing of Genomes and Proteomes

All genomic annotations and proteomes as both amino acid and DNA were downloaded from the NCBI Refseq FTP site. Orphans were detected as previously described [4] using NCBI Blast [50] and a cutoff of 10^−3^ and then loaded into the *OrphanMine* database for post-processing. The *OrphanMine* interface was used to generate groups of TRGs for each taxonomic level. A custom Perl script was used to calculate length, G+C content and cost and to parse blast reports to generate a “neighbourhood distribution” (ND) for each CDS. All of the data used in this study is publicly available through the *OrphanMine*. The code used to generate lists of orphans from proteomes is available in the YAMAP package (www.genomics.ceh.ac.uk/yamap/) and all other code (any additional Perl scripts) is available on request (gawi@ceh.ac.uk).

### Calculation of QIPP scores

For each genome and for each of the five selected criteria, the distribution of non-orphans was generated and the percentiles for that distribution were calculated. For the criteria of length and ND, the absolute value of each component criterion (e.g. length of 200 amino acids) was transformed into a sub-score from 0 to 100 depending on the percentile in which it fell (e.g. the 35^th^ percentile from the shortest CDS found would be given a score of 35). For low complexity and cost, where more of either actually suggests a less probable CDS, the score was subtracted from 100 (e.g. a protein with 50% low complexity might fall in the 70^th^ percentile and therefore be given a low score of 30). G+C content had to be calculated as the deviation from the mean value. Values above the 50th percentile were corrected by the equation 100 minus the percentile value multiplied by two and values below had their percentile doubled.

Length was calculated as the total number of amino acids and percentage low complexity regions was calculated from regions masked with the seg program [21] using default parameters. G+C content was calculated from the proteome as DNA. The average amino acid cost of a sequence was calculated using the relative costs for each amino acid according to the values given in Akashi & Gojobori [23]. Randomized proteomes (*i.e.* any sequence evolving neutrally) are the most costly as purifying selection appears to select for amino acids that are less metabolically expensive [23]. ND was calculated by determining the level of conservation of the five flanking CDS on either side of a particular CDS. For each of these ten genes, the number of species in which a similar sequence was found was recorded (maximum of 121 for this dataset). Those numbers were then summed, averaged and percentiles generated for the distribution.

The scores from all five criteria are normalized with respect to each particular genome and can therefore be summed. To obtain a final QIPP score between zero and 1, the average is taken and divided by 100. Zero would be the worst possible candidate for a real gene while 1 would be ideal. Using the interface to the *OrphanMine* it is possible to perform user-selected rankings of subsets of the CDS held in the database on the basis of 1 or all of the component criteria used in QIPP. To compare QIPP and Glimmer scores, the five reference genomes were run through Glimmer (v2.13) [51] with default settings.

### Genetic Similarity of genomes and the taxonomic distribution of TRGs

The Index of Isolation of an Organism (IIO) similarity measure was calculated by averaging the logarithm of the best e-value for each CDS in a proteome as described by Fukuchi & Nishikawa [8]. The taxonomic distribution of each CDS in the five references genomes (Table 3) was obtained through interrogation of the *OrphanMine* database [4]. For each genome, appropriate queries were performed to find genes restricted to each taxonomic level. The output was scored and downloaded in a tab-delimited format. A Perl script was written to parse the output to ensure that every predicted protein was only counted once and each protein could be classed according to its lineage specificity.

### Obtaining Empirical Data from Microarray and Proteomic Studies

The MicrobesOnline database [27] was queried for the *E.coli* orphan genes using their unique VIMSS ID. A file was provided by Keith Keller to map the GenBank IDs of the orphan genes obtained from *OrphanMine* to the VIMSS ID. EchoBASE is a database that curates information regarding the genes and gene products of the model bacterium *E. coli* K-12, including links to literature describing proteomic analyses of this bacterium [*52*]. The ‘b number’ identifiers provided in the literature were used to map data from the proteomic analyses to the *E.coli* orphan genes obtained from *OrphanMine*. When ‘b numbers’ were not provided, the gene name, if present, was used.

## Supporting Information

Table S1 The number of predicted proteins, orphans, percentage orphans, isolation index and taxonomic uniqueness for each of the 122 bacterial genomes used in this analysis
(0.22 MB DOC)

Figure S1 Relationship between the numbers of orphans as a percentage of total predicted proteins and Isolation Index of an Organism. The IIO for each genome in our dataset (full list of genomes given in Table S1) is plotted against (a) percentage of orphans, (b) the number of orphans greater than 200 aa's and (c) the percentage of total orphans greater than 200 aa's in length. In addition, each genome is classed according to the taxonomic level at which it is the only sequenced representative.
(3.29 MB TIF)
