# Supplementary material for: Large-Scale Comparative Genomic Ranking of Taxonomically Restricted Genes (TRGs) in Bacterial and Archaeal Genomes
Source: PLoS One. 2007 Mar 28;2(3):e324. doi: 10.1371/journal.pone.0000324 (PMC1824705; doi:10.1371/journal.pone.0000324)
Supplement: Table S1 — The number of predicted proteins, orphans, percentage orphans, isolation index and taxonomic uniqueness for each of the 122 bacterial genomes used in this analysis (0.22 MB DOC) [file pone.0000324.s001.doc]

**Table S1. The number of predicted proteins, orphans, percentage orphans, isolation index and taxonomic uniqueness for each of the 122 bacterial genomes used in this analysis**

| Species | Predicted Proteins | Orphans | % Orphans | IIO | Taxonomic Uniqueness |
| --- | --- | --- | --- | --- | --- |
| Haemophilus influenzae Rd | 1657 | 38 | 2.29 | -218.02 | Species |
| Mycoplasma genitalium | 484 | 2 | 0.41 | -233 | Species |
| Synechocystis sp PCC6803 | 3167 | 223 | 7.04 | -153.99 | Genus |
| Methanococcus jannaschii | 1729 | 259 | 14.98 | -103.83 | Family |
| Mycoplasma pneumoniae | 689 | 67 | 9.87 | -188.09 | Species |
| Helicobacter pylori 26695 | 1576 | 261 | 16.56 | -125.97 | Species |
| Escherichia coli K12 | 4311 | 174 | 4.04 | -259.98 | Genus |
| Methanobacterium thermoautotrophicum Delta H | 1873 | 293 | 15.64 | -104.14 | Family |
| Bacillus subtilis,subsp. subtilis str. 168 | 4112 | 460 | 11.19 | -150.05 | Species |
| Archaeoglobus fulgidus | 2420 | 391 | 16.16 | -95.47 | Family |
| Borrelia burgdorferi | 851 | 152 | 17.86 | -93.49 | Genus |
| Aquifex aeolicus | 1529 | 138 | 9.03 | -108.15 | Division |
| Pyrococcus horikoshii | 1956 | 180 | 9.2 | -205.47 | Species |
| Mycobacterium tuberculosis H27Rv | 3927 | 13 | 0.33 | -294.93 | Species |
| Treponema pallidum | 1036 | 248 | 23.94 | -86.01 | Genus |
| Chlamydia trachomatis D/UW-3/CX | 895 | 46 | 5.14 | -209.85 | Species |
| Rickettsia prowazekii | 835 | 19 | 2.28 | -253.82 | Species |
| Chlamydophila pneumoniae CWL029 | 1054 | 102 | 9.68 | -200.75 | Species |
| Aeropyrum pernix | 1841 | 499 | 27.1 | -78.21 | Family |
| Thermotoga maritima | 1858 | 224 | 12.06 | -108.7 | Division |
| Deinococcus radiodurans | 2997 | 591 | 19.72 | -87.47 | Division |
| Campylobacter jejuni | 1634 | 161 | 9.85 | -134.52 | Family |
| Neisseria meningitidis MC58 | 2079 | 434 | 20.88 | -138.93 | Genus |
| Bacillus halodurans | 4066 | 456 | 11.22 | -143.81 | Species |
| Xylella fastidiosa | 2766 | 824 | 29.79 | -146.52 | Genus |
| Vibrio cholerae | 3835 | 504 | 13.14 | -200.1 | Species |
| Buchnera sp. APS | 564 | 2 | 0.35 | -234 | Species |
| Thermoplasma acidophilus | 1482 | 55 | 3.71 | -197.13 | Genus |
| Pseudomonas aeruginosa PAO1 | 5567 | 306 | 5.5 | -198.07 | Species |
| Ureaplasma urealyticum | 614 | 117 | 19.06 | -98.92 | Genus |
| Halobacterium sp. NRC-1 | 2075 | 485 | 23.37 | -75.64 | Family |
| Mesorhizobium loti | 6746 | 864 | 12.81 | -146.7 | Family |
| Thermoplasma volcanium | 1499 | 53 | 3.54 | -193.65 | Species |
| Mycobacterium leprae | 1605 | 89 | 5.55 | -245.57 | Species |
| Pasteurella multocida Pm70 | 2015 | 83 | 4.12 | -212.23 | Genus |
| Streptococcus pyogenes M1GAS | 1697 | 147 | 8.66 | -189.56 | Species |
| Staphylococcus aureus subsp. aureus N315 | 2593 | 205 | 7.91 | -192.07 | Species |
| Lactococcus lactis subsp. lactis | 2321 | 336 | 14.48 | -136.45 | Genus |
| Mycoplasma pulmonis | 782 | 149 | 19.05 | -89.79 | Species |
| Caulobacter crescentus | 3737 | 475 | 12.71 | -128.28 | Family |
| Sulfolobus solfataricus | 2977 | 271 | 9.1 | -148.85 | Species |
| Streptococcus pneumoniae TIGR4 | 2094 | 330 | 15.76 | -158.42 | Species |
| Sinorhizobium meliloti | 3341 | 140 | 4.19 | -198.6 | Genus |
| Clostridium acetobutylicum | 3672 | 556 | 15.14 | -127.15 | Species |
| Sulfolobus tokodaii | 2826 | 426 | 15.07 | -138.95 | Species |
| Rickettsia conorii Malish 7 | 1374 | 354 | 25.76 | -159.38 | Species |
| Yersinia pestis C092 | 3885 | 325 | 8.37 | -197.25 | Genus |
| Salmonella enterica subsp. enterica serovar Typhi | 4395 | 173 | 3.94 | -263.64 | Species |
| Salmonella typhimurium LT2 | 4451 | 97 | 2.18 | -269.58 | Species |
| Listeria innocua | 2968 | 145 | 4.89 | -242.27 | Species |
| Listeria monocytogenes | 2846 | 76 | 2.67 | -252.97 | Species |
| Nostoc sp PCC7120 (Cyanobacteria) | 5366 | 892 | 16.62 | -128.15 | Family |
| Agrobacterium tumefaciens (C58 Cereon) | 4554 | 356 | 7.82 | -184.73 | Genus |
| Brucella melitensis 16M | 3198 | 284 | 8.88 | -176.83 | Family |
| Clostridium perfringens 13 | 2660 | 285 | 10.71 | -144.81 | Species |
| Pyrobaculum aerophilum | 2605 | 945 | 36.28 | -62.39 | Family |
| Ralstonia solanacearum GM1000 | 3440 | 399 | 11.6 | -146.95 | Family |
| Pyrococcus furiosus DSM 3638 | 2125 | 142 | 6.68 | -190.17 | Species |
| Pyrococcus abyssi | 1896 | 59 | 3.11 | -217.21 | Species |
| Corynebacterium glutamicum ATCC 13032 | 2993 | 304 | 10.16 | -196.51 | Species |
| Methanopyrus kandleri AV19 | 1687 | 399 | 23.65 | -83.63 | Family |
| Fusobacterium nucleatum subsp. nucleatum ATCC 25586 | 2067 | 335 | 16.21 | -106.12 | Division |
| Methanosarcina acetivorans str. C2A | 4540 | 695 | 15.31 | -174.12 | Species |
| Thermoanaerobacter tengcongensis | 2588 | 335 | 12.94 | -120.74 | Family |
| Streptomyces coelicolor A3(2) | 7769 | 700 | 9.01 | -187.56 | Species |
| Xanthomonas campestris pv. campestris str. ATCC 33913 | 4181 | 159 | 3.8 | -249.12 | Species |
| Xanthomonas axonopodis pv. citri str. 306 | 4312 | 239 | 5.54 | -242.74 | Species |
| Buchnera aphidicola str. Sg (Schizaphis graminum) | 546 | 1 | 0.18 | -233.82 | Species |
| Chlorobium tepidum TLS | 2252 | 545 | 24.2 | -97.83 | Family |
| Methanosarcina mazei Goe1 | 3371 | 237 | 7.03 | -206.07 | Species |
| Thermosynechococcus elongatus BP-1 | 2475 | 165 | 6.67 | -163.89 | Genus |
| Streptococcus agalactiae 2603V/R | 2124 | 235 | 11.06 | -177.09 | Species |
| Oceanobacillus iheyensis HTE 831 | 3500 | 310 | 8.86 | -147.23 | Genus |
| Shewanella oneidensis MR-1 | 4324 | 602 | 13.92 | -138.47 | Family |
| Shigella flexneri 2a str. 301 | 4180 | 110 | 2.63 | -256.23 | Genus |
| Wigglesworthia brevipalpis | 611 | 4 | 0.65 | -177.01 | Genus |
| Bifidobacterium longum NCC2705 | 1727 | 206 | 11.93 | -120.13 | Family |
| Streptococcus mutans UA159 | 1960 | 198 | 10.1 | -174.91 | Species |
| Mycoplasma penetrans HF-2 | 1037 | 237 | 22.85 | -88.72 | Species |
| Pseudomonas putida KT2440 | 5350 | 428 | 8 | -202.63 | Species |
| Vibrio vulnificus CMCP6 | 4537 | 363 | 8 | -210.89 | Species |
| Bradyrhizobium japonicum USDA 110 | 8317 | 1060 | 12.75 | -155.74 | Genus |
| Staphylococcus epidermis ATCC 12228 | 2419 | 228 | 9.43 | -193.8 | Species |
| Chlostridium tetani Massachusetts E88 | 2373 | 176 | 7.42 | -156.36 | Species |
| Lactobacillus plantarum WCFS1 | 3009 | 408 | 13.56 | -126.17 | Family |
| Tropheryma whipplei TW08/27 | 783 | 91 | 11.62 | -116.16 | Family |
| Vibrio parahaemolyticus RIMD 2210633 | 4832 | 634 | 13.12 | -199.02 | Species |
| Bacteroides thetaiotaomicron VPI-5482 | 4778 | 1082 | 22.65 | -104.35 | Family |
| Enterococcus faecalis V583 | 3113 | 546 | 17.54 | -130.73 | Family |
| Streptomyces avermitilis MA-4680 | 7575 | 671 | 8.86 | -191.34 | Species |
| Chlamydophila caviae GPIC | 998 | 66 | 6.61 | -211.69 | Species |
| Leptospira interrogans serovar lai str. 56601 | 4727 | 2138 | 45.23 | -54.55 | Family |
| Coxiella burnetii RSA 493 | 2009 | 649 | 32.3 | -98.66 | Family |
| Nitrosomonas europaea ATCC 19718 | 2461 | 227 | 9.22 | -141.87 | Family |
| Bacillus cereus ATCC 14579 | 5234 | 288 | 5.5 | -219.54 | Species |
| Bacillus anthracis Ames | 5311 | 471 | 8.87 | -213.99 | Species |
| Mycobacterium bovis AF2122/97 (spoligotype 9) | 3920 | 22 | 0.56 | -292.39 | Species |
| Helicobacter hepaticus ATCC51449 | 1875 | 368 | 19.63 | -129.07 | Species |
| Corynebacterium efficiens YS-314T | 2950 | 289 | 9.8 | -198.89 | Species |
| Pirellula sp. 1 | 7325 | 3576 | 48.82 | -49.79 | Division |
| Haemophilus ducreyi 35000HP | 1717 | 284 | 16.54 | -164 | Species |
| Candidatus Blochmannia floridanus | 583 | 1 | 0.17 | -193.26 | Genus |
| Bordetella pertussis Tohama I NCTC-13251 | 3447 | 7 | 0.2 | -290.4 | Species |
| Bordetella parapertussis 12822 NCTC-13253 | 4185 | 14 | 0.33 | -306.04 | Species |
| Bordetella bronchiseptica RB50 NCTC-13252 | 4994 | 117 | 2.34 | -283.52 | Species |
| Prochlorococcus marinus CCMP1375(SS120) | 1882 | 291 | 15.46 | -155.63 | Family |
| Synechococcus sp.WH8102 | 2517 | 388 | 15.42 | -139.08 | Genus |
| Mycoplasma gallisepticum R | 726 | 76 | 10.47 | -108.29 | Species |
| Pseudomonas syringae pv. Tomato DC3000 | 5471 | 573 | 10.47 | -194.24 | Species |
| Porphyromonas gingivalis W83 | 1909 | 352 | 18.44 | -137.14 | Family |
| Chromobacterium violaceum ATCC 12472 | 4407 | 577 | 13.09 | -138.63 | Genus |
| Wolinella succinogenes | 2044 | 150 | 7.34 | -138.64 | Genus |
| Photorhabdus luminescens laumondii TT01 | 4683 | 719 | 15.35 | -155.29 | Genus |
| Gloeobacter violaceus PCC7421 | 4430 | 682 | 15.4 | -115.08 | Family |
| Nanoarchaeum equitans Kin4-M | 563 | 167 | 29.66 | -64.64 | Division |
| Corynebacterium diphtheriae gravis NCTC13129 | 2272 | 259 | 11.4 | -168.21 | Species |
| Geobacter sulfurreducens PCA | 3445 | 580 | 16.84 | -106.03 | Family |
| Rhodopseudomonas palustris CGA009 | 4814 | 336 | 6.98 | -191.98 | Genus |
| Phytoplasma asteris OY | 754 | 229 | 30.37 | -67.1 | Family |
| Bdellovibrio bacteriovorus HD100 | 3583 | 1113 | 31.06 | -73.66 | Family |
| Mycobacterium avium,subsp.:paratuberculosis K-10 | 4350 | 211 | 4.85 | -200.77 | Species |
| Mycoplasma mycoides,subsp.mycoides SC | 1016 | 209 | 20.57 | -85.65 | Species |
