# Supplementary figures and images for: Large-Scale Comparative Genomic Ranking of Taxonomically Restricted Genes (TRGs) in Bacterial and Archaeal Genomes
Source: PLoS One. 2007 Mar 28;2(3):e324. doi: 10.1371/journal.pone.0000324 (PMC1824705; doi:10.1371/journal.pone.0000324)

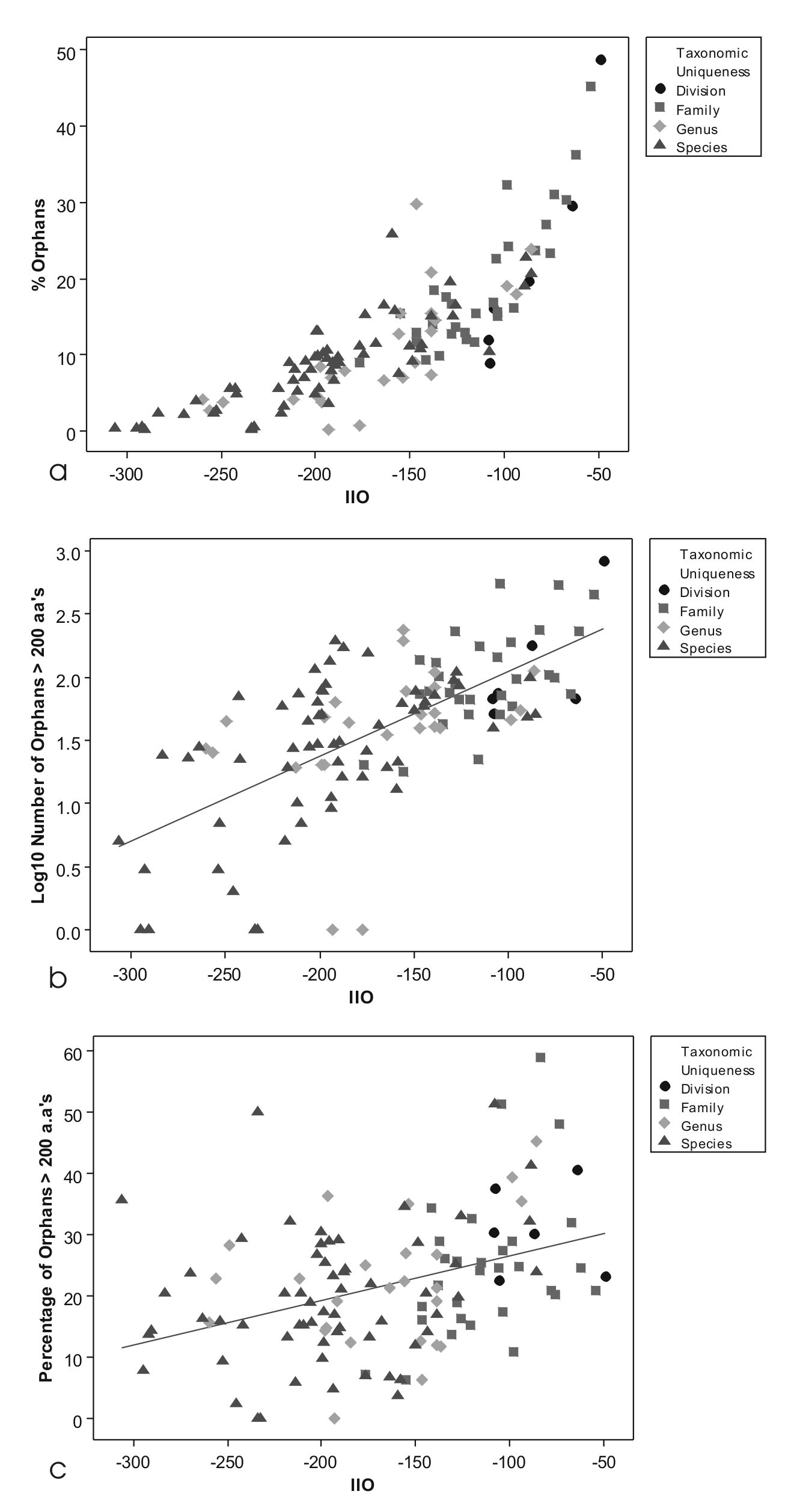

Supplement: Figure S1 — Relationship between the numbers of orphans as a percentage of total predicted proteins and Isolation Index of an Organism. The IIO for each genome in our dataset (full list of genomes given in Table S1) is plotted against (a) percentage of orphans, (b) the number of orphans greater than 200 aa's and (c) the percentage of total orphans greater than 200 aa's in length. In addition, each genome is classed according to the taxonomic level at which it is the only sequenced representative. (3.29 MB TIF) [file pone.0000324.s002.tif]
